# Rapamycin and WYE-354 suppress human gallbladder cancer xenografts in mice

**DOI:** 10.18632/oncotarget.5047

**Published:** 2015-09-11

**Authors:** Helga Weber, Pamela Leal, Stefan Stein, Hana Kunkel, Patricia García, Carolina Bizama, Jaime A. Espinoza, Ismael Riquelme, Bruno Nervi, Juan C. Araya, Manuel Grez, Juan C. Roa

**Affiliations:** ^1^ Department of Pathology, Center of Genetic and Immunological Studies (CEGIN) and Scientific and Technological Bioresource Nucleus (BIOREN), Universidad de La Frontera, Temuco, Chile; ^2^ Gene Therapy Unit, Institute for Tumor Biology and Experimental Therapy, Georg-Speyer-Haus, Frankfurt, Germany; ^3^ Department of Pathology, UC-Center for Investigational Oncology (CITO), Advanced Center for Chronic Diseases (ACCDiS), School of Medicine, Pontificia Universidad Católica de Chile, Santiago, Chile; ^4^ Department of Hematology Oncology, UC-Center for Investigation in Translational Oncology (CITO), School of Medicine, Pontificia Universidad Católica de Chile, Santiago, Chile

**Keywords:** gallbladder cancer, mTOR inhibitors, gallbladder cancer xenografts, rapamycin, WYE-354

## Abstract

Gallbladder cancer (GBC) is a highly malignant tumor characterized by a poor response to chemotherapy and radiotherapy. We evaluated the *in vitro* and *in vivo* antitumor efficacy of mTOR inhibitors, rapamycin and WYE-354. *In vitro* assays showed WYE-354 significantly reduced cell viability, migration and invasion and phospho-P70S6K expression in GBC cells. Mice harboring subcutaneous gallbladder tumors, treated with WYE-354 or rapamycin, exhibited a significant reduction in tumor mass. A short-term treatment with a higher dose of WYE-354 decreased the tumor size by 68.6% and 52.4%, in mice harboring G-415 or TGBC-2TKB tumors, respectively, compared to the control group. By contrast, treatment with a prolonged-low-dose regime of rapamycin almost abrogated tumor growth, exhibiting 92.7% and 97.1% reduction in tumor size, respectively, compared to control mice. These results were accompanied by a greater decrease in the phosphorylation status of P70S6K and a lower cell proliferation Ki67 index, compared to WYE-354 treated mice, suggesting a more effective mTOR pathway inhibition. These findings provide a proof of concept for the use of rapamycin or WYE-354 as potentially good candidates to be studied in clinical trials in GBC patients.

## INTRODUCTION

Gallbladder cancer (GBC) is the most common type of biliary-tract carcinomas [1]. Most cases are diagnosed in advanced stages and patients cannot undergo curative surgery. Chemotherapy has been the main treatment option with agents such as gemcitabine, cisplatin, oxaliplatin, capecitabine, and 5- fluorouracil (5-FU) [2, 3]. A recent randomized phase III clinical trial (ABC-02 trial) established the cisplatin/gemcitabine (Gem/CDDP) combination as standard treatment regimen for patients with advanced biliary-tract cancer [4, 5]. However, the response to chemotherapy and radiotherapy is extremely limited, with modest impact in overall survival [4, 6–12].

The recent progress in identification and understanding of molecular alterations of GBC may improve the clinical management of patients through the application of more specific and effective therapies [11, 13, 14]. The phosphoinositide 3-kinase (PI3K)/mammalian target of rapamycin (mTOR) signaling pathway has been proposed as an interesting therapeutic target in cancer with a pivotal role in cell cycle progression, cell proliferation and angiogenesis [15–30]. This pathway is frequently activated in different human cancers by a variety of genetic and epigenetic events [31]. Molecular therapies targeting mTOR, have being actively investigated and many of these have now progressed to clinical trials in many different types of cancer [16, 17, 32].

Previous studies by our group have shown the upregulation of the AKT/mTOR signaling pathway in advanced GBC. Through immunohistochemical assays, we have found that phospho-mTOR and phospho-P70S6K expression are significantly elevated in human gallbladder carcinoma compared to non-neoplastic tissues [33, 34]. Moreover, high phospho-mTOR expression was correlated with a worse prognosis in patients with advanced GBC [34]. *In vitro* analysis in GBC cell lines have demonstrated the efficacy of mTOR inhibitors on reducing cell growth, cell migration, and phospho-P70S6K expression [33, 34]. Preclinical studies also have confirmed the therapeutic effects of mTOR inhibitors. Wu *et al*., have reported that rapamycin can reduce the incidence of gallbladder carcinoma in BK5.erbB2 transgenic mouse models [35]. Zong *et al*., reported that the mTOR pathway inhibition attenuates the migration and invasion capacities of GBC using *in vivo* tumor metastasis mouse model [36]. These findings provide a rationale for the potential use of mTOR inhibitors as a therapeutic strategy for human gallbladder carcinoma.

Rapamycin is an mTOR inhibitor and an antifungal agent with immunosuppressive properties, which has an established effect on suppressing tumor growth in a number of solid tumors [37]. The efficiency of mTOR inhibitors has enabled the development of a number of rapalogs (rapamycin analogs). These rapalogs, which include sirolimus, temsirolimus, everolimus, and deforolimus [15], are allosteric inhibitors of mTORC1 that form a complex with the intracellular receptor FKBP12, which binds to mTOR and inhibits mTORC1 downstream signaling. Apart from rapalogs, there is now strong interest in small-molecule ATP-competitive inhibitors of mTOR kinase, which can either act selectively on mTORC1 and mTORC2 (AZD8055, WYE-354, PP30, PP242) or as dual PI3K- and mTOR inhibitors (BEZ235 and XL765) [18, 38].

In this study we evaluated the antitumor activity of the allosteric mTORC1 inhibitor, rapamycin and of the ATP-competitive mTOR inhibitor WYE-354 on preclinical xenograft GBC tumor models.

## RESULTS

### WYE-354 reduces cell viability and phosphorylation of AKT/mTOR downstream proteins in gallbladder cancer cells

We decided to study the *in vitro* effect of an ATP-competitive mTOR inhibitor, WYE-354, which inhibits the catalytic activity of mTORC1 and mTORC2, unlike rapamycin and its rapalogs, which are specific inhibitors of mTORC1 [21, 38]. In an earlier publication of our group, rapamycin also has been shown to significantly decrease cell viability in gallbladder cancer cells [33].

**Figure 1:**
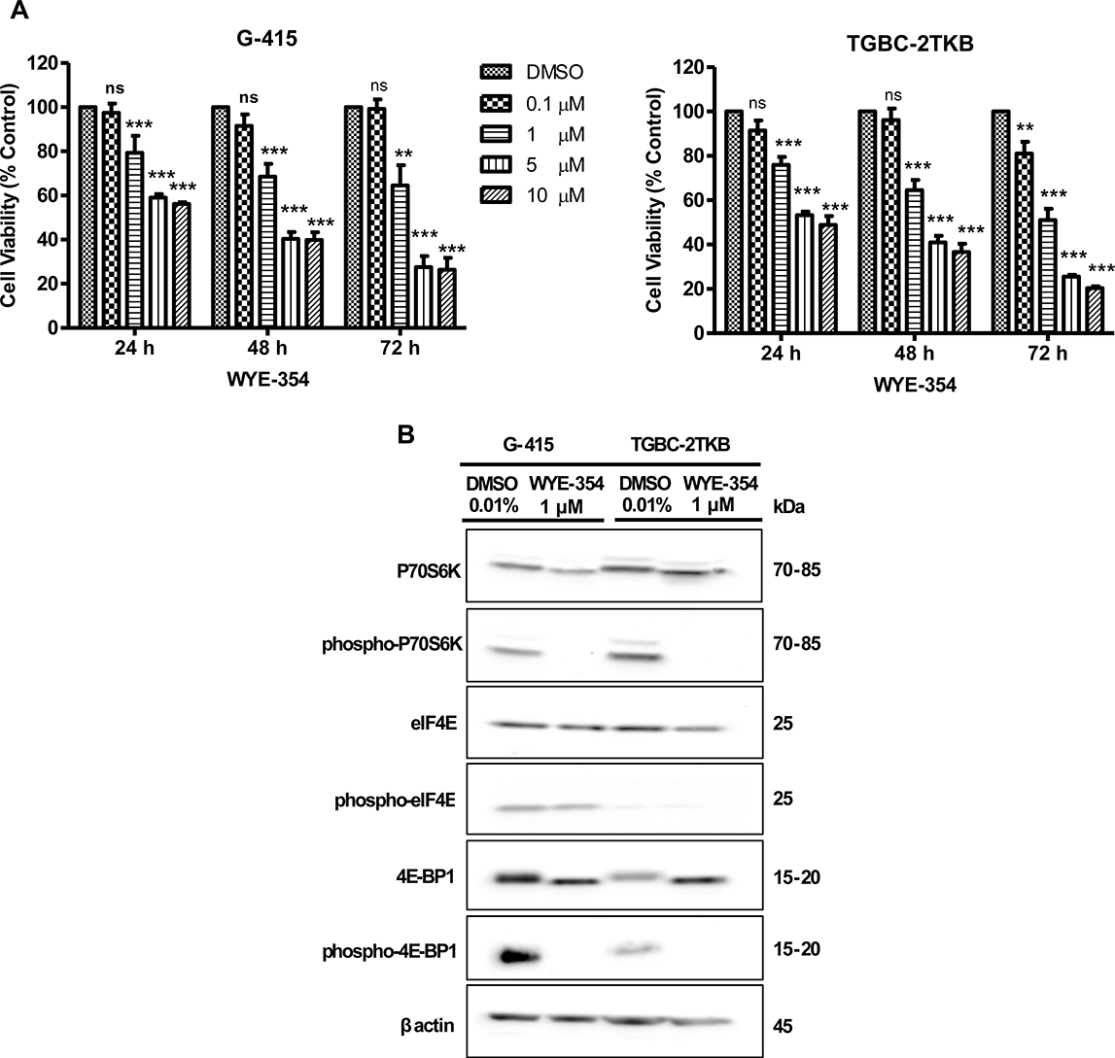
*In vitro* effects of WYE-354 on cell growth and mTOR signaling pathway in two gallbladder cancer cell lines **A.** G-415 and TGBC-2TKB cells were treated with increasing concentrations of WYE-354. Cell viability was determined after 24, 48, and 72 hours of treatment. Data are shown as mean ± SD of at least three independent experiments in quintuplicate (**P < 0.01; ***P < 0.001; ns: not significant). **B.** G-415 and TGBC-2TKB cells were treated with WYE-354 (1 μM), for 18 hours. Control cells received an equivalent amount of solvent (0.01% dimethylsulfoxide). Western blot analysis was carried out using antibodies against the total and phosphorylated portion of P70S6K, 4E-BP1, and eIF4E proteins. Protein loading was normalized using an antibody recognizing β-actin.

Cell viability was analyzed by MTS assay according to the manufacturer's protocol. Cells were treated with increasing concentrations of WYE-354 for 24, 48, and 72 hours. As shown in Figure 1A, WYE-354 significantly reduced cell viability starting at a 1 μM concentration after a 24 hours exposure, in both studied cell lines (*P* < 0.001). We did not observe a decrease in cell viability at a dose of 100 nM, except for the TGBC-2TKB cell line after 72 hours of treatment.

To further investigate the *in vitro* effects of WYE-354 on mTOR signaling, we evaluated the phosphorylation status of mTOR effectors by immunoblotting. Cells were exposed to WYE-354 (1 μM) and 0.01% dimethylsulfoxide (as control) for 18 hours and were lysed and analyzed by Western blot using commercial antibodies. As shown in Figure 1B, the phosphorylation of the downstream effectors of mTOR, 4E-BP1 and P70S6K, were strongly inhibited in *vitro* by WYE-354 treatment. No significant changes were observed in phospho-eIF4E and in total P70S6K, 4E-BP1 and eIF4E protein expression under the treatment conditions assayed.

### Exposure to mTOR inhibitors decreases *in vitro* cell migration and invasion in gallbladder cancer cells

**Figure 2:**
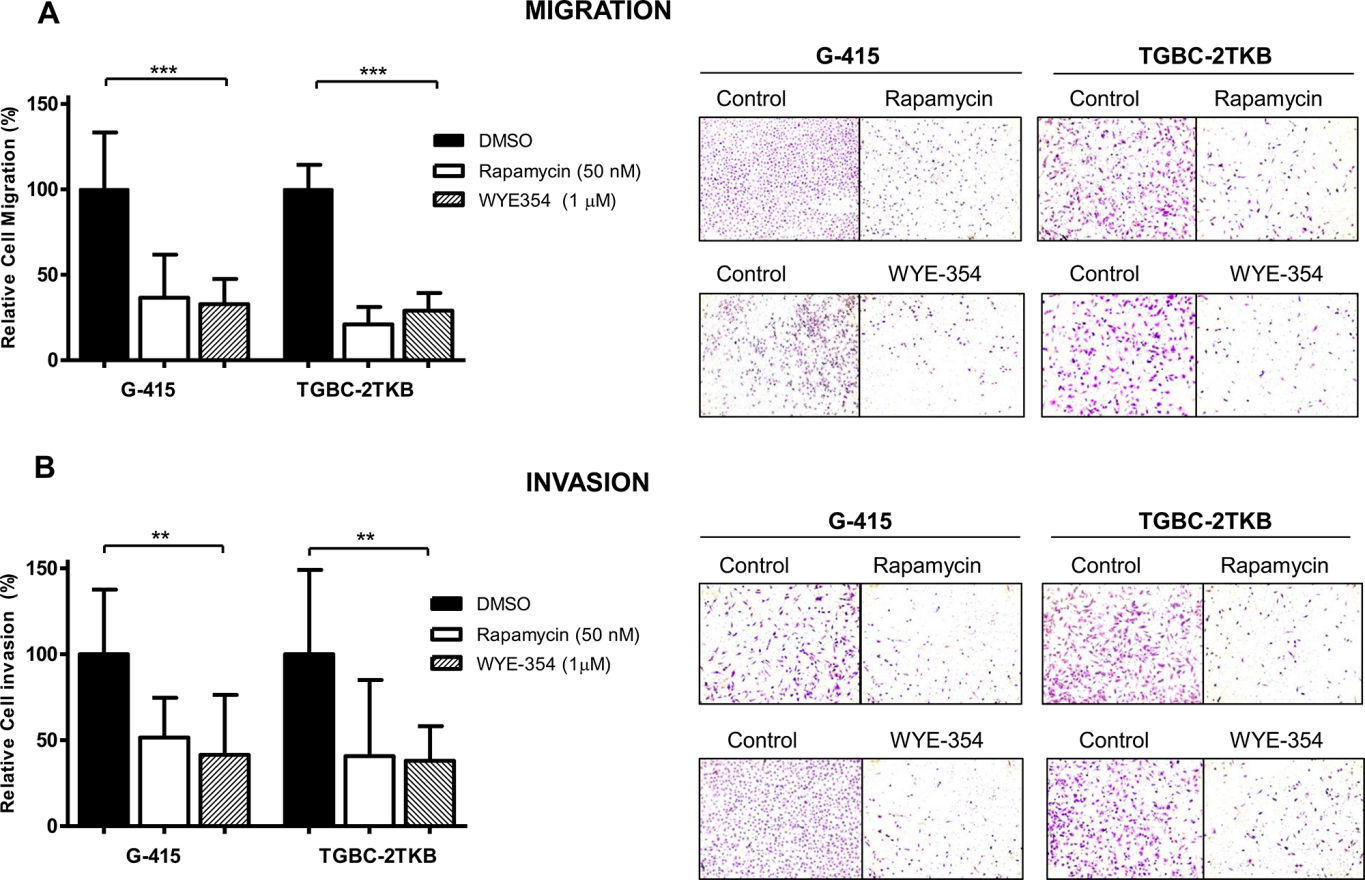
Effect of WYE-354 and rapamycin on cell migration and invasion **A.** Migration and **B.** invasion assays were performed using 24-well transwell plates containing polycarbonate filters with an 8 μm pore size. Before seeding, G-415 and TGBC-2TKB cells were exposed to WYE-354 (1 μM), rapamycin (50 nM) or dimethylsulfoxide (0.01%) (control) for 12 hours at 37°C. Cells were counted in six randomly selected fields after 24 hours. Results are expressed as mean ± SD (**P < 0.001; ***P < 0.001).

In order to establish the effect on cell migration and cell invasion of WYE-354 and rapamycin, G-415 and TGBC-2TKB were exposed to 0.01% dimethylsulfoxide (as control), WYE-354 (1 μM), or rapamycin (50 nM) for 12 hours. After 24 hours, the migration and invasion rates were significantly lower in treated cells compare with untreated cells (*P* < 0.001; *P* < 0.01, respectively). Relative migration rates observed in G-415 were 36.7% (rapamycin) and 32.8% (WYE-354), while TGBC-2TKB showed a migration rate of 21.0% (rapamycin) and 28.9% (WYE-354) (Figure 2A). Relative invasion rates in G-415 cells were 51.6% (rapamycin) and 41.5% (WYE-354), while TGBC-2TKB exhibited 41.0% (rapamycin) and 38.1% (WYE-354) (Figure 2B).

### Rapamycin and WYE-354 inhibit tumor growth on xenograft gallbladder cancer model

**Figure 3:**
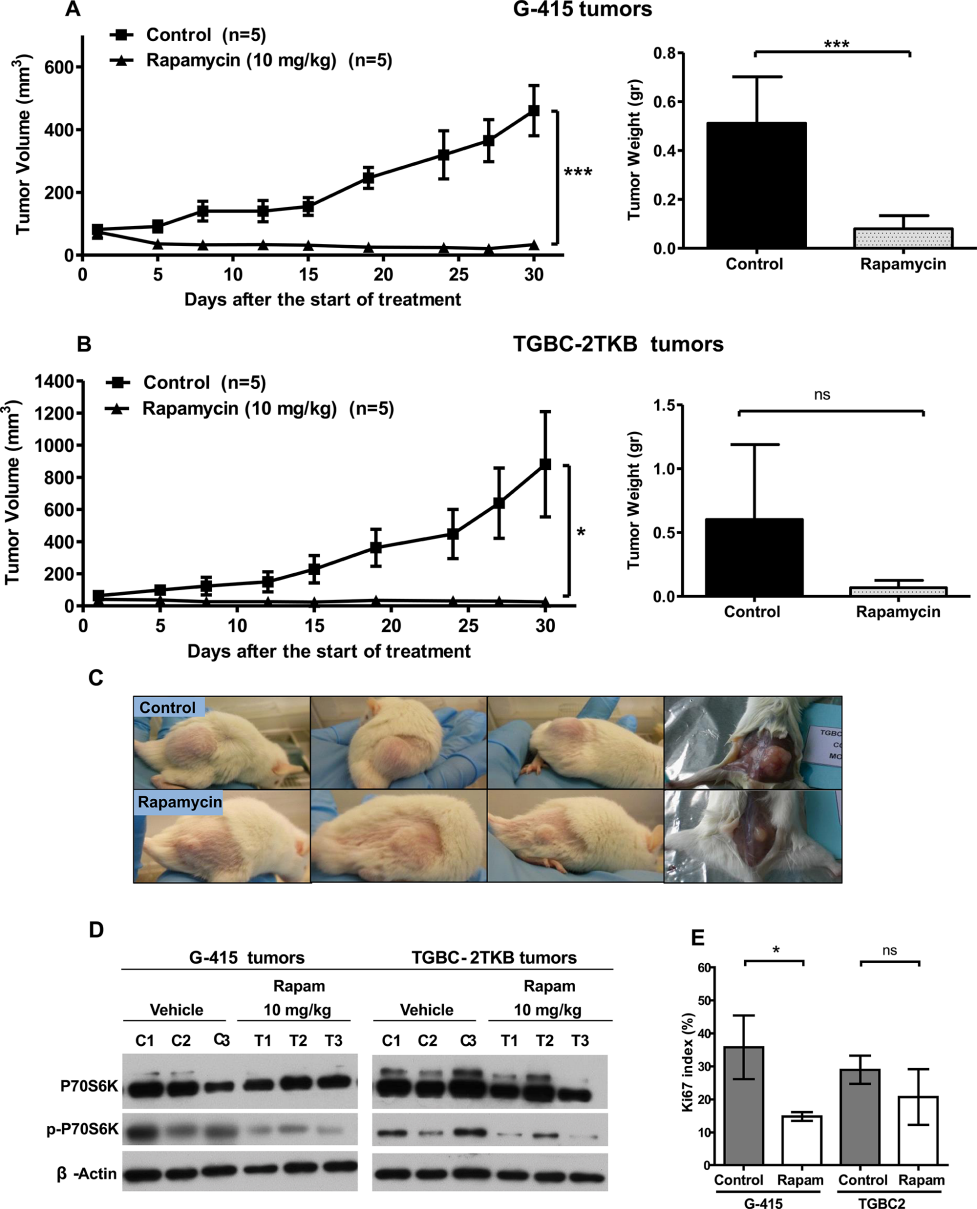
*In vivo* efficacy of rapamycin on human gallbladder cancer xenografts **A.** and **B.** Tumor growth of mice harboring G-415 or TGBC2TKB tumors. G-415 or TGBC2TKB cells were injected subcutaneously into NOD-SCID mice. When tumors reached a volume of approximately 100 mm^3^, they were treated with rapamycin or vehicle, as described in the Materials and Methods section. Animals were sacrificed when tumors reached approximately 1500 mm^3^ or at day 30. Data are expressed as mean ± SD. Rapamycin exerted a statistically significant antitumor effect (compared with the groups treated with the vehicle (*P < 0.05; ***P < 0.001 at day 30)). **C.** Representative photographs of mice treated with vehicle or rapamycin. **D.** Western blot analysis of total P70S6K and phospho-P70S6K of tumor tissue from mice treated with vehicle or rapamycin. **E.** Ki67 proliferation index was assessed by immunohistochemistry in G-415 and TGBC-2TKB xenografts collected 30 days after starting the treatment. Ki67 positive cells were counted to calculate the Ki67 proliferation index. Data are expressed as mean ± SD. (*P < 0.05; ns: not significant).

**Figure 4:**
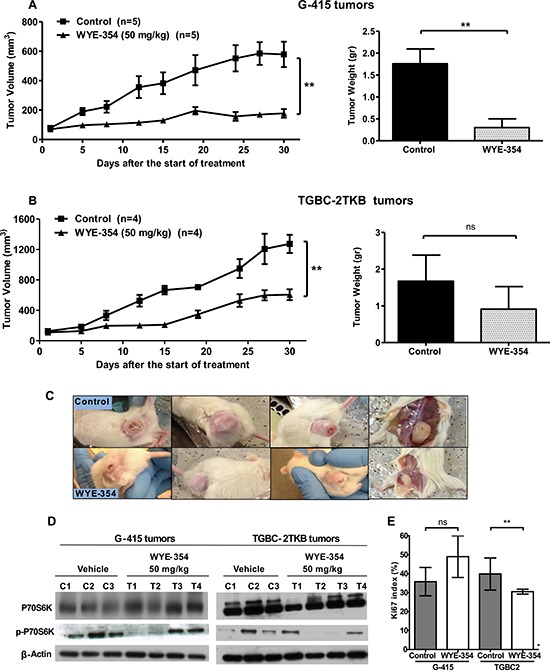
*In vivo* efficacy of WYE-354 on human gallbladder cancer xenografts **A.** and **B.** Tumor growth of mice harboring G-415 or TGBC2TKB tumors. G-415 or TGBC2TKB cells were injected subcutaneously into NOD-SCID mice. When tumors reached a volume of approximately 100 mm^3^, they were treated with vehicle or WYE-354, as described in the Materials and Methods section. Animals were sacrificed when tumors reached approximately 1500 mm^3^ or at day 30. Data are expressed as mean ± SD. WYE-354 exerted a statistically significant antitumor effect (compared with the groups treated with the vehicle (**P < 0.01 at day 30)). **C.** Representative photographs of mice treated with vehicle or WYE-354. **D.** Western blot analysis of total P70S6K and phospho-P70S6K of tumor tissue from mice treated with vehicle or WYE-354. **E.** Ki67 proliferation index was assessed by immunohistochemistry in G-415 and TGBC-2TKB xenografts collected 30 days after the starting the treatment. Ki67 positive cells were counted to calculate the Ki67 proliferation index. Data are expressed as mean ± SD. (**P < 0.01; ns: not significant).

Based on the above information and previous work, we decided to study further whether mTOR inhibitors can be therapeutically effective *in vivo* on subcutaneously established human gallbladder tumors. The effect of rapamycin and WYE-354 on tumor growth was evaluated in xenograft GBC tumor models. 2 × 10^6^ or 5 × 10^6^ cells of G-415 or TGBC2TKB, respectively, were xenotransplanted into NOD-SCID mice subcutaneously. When tumors reached an average volume of 100 mm^3^, the mice were treated either with rapamycin or WYE354. Rapamycin was administered i.p. at a concentration of 10 mg/kg, daily for 5 days per week for 3 weeks, while WYE-354 was administrated at a daily i.p. dose of 50 mg/kg for 5 days. Mice were sacrificed 30 days after the initiation of the treatments and an autopsy was performed that included removal of the entire tumor area. Mice bearing G-415 or TGBC-2TKB tumors and treated with rapamycin exhibited 92.7% and 97.1% reduction in average tumor size (*P* < 0.001; *P* < 0.5), as well as 84.3% and 88.7% in tumor weight (*P* < 0.001; ns) compared to the control, respectively (Figures 3A, 3B and 3C). While mice treated with WYE-354 exhibited 68.6% and 52.4% reduction in average tumor size (*P* < 0.01; *P* < 0.01), as well as 82.9% and 45.5% (*P* < 0.01; ns) reduction in tumor weight, respectively. (Figures 4A, 4B and 4C). Macroscopic analysis of the tumors revealed decreased vascularization in treated groups compared to the control groups. This effect was stronger in mice treated with rapamycin. Inhibition of mTOR signaling by rapamycin and WYE-354 in tumor tissues obtained at the end of the *in vivo* studies was assessed by Western blot analysis. As shown in Figures 3D and 4D, treatment with either mTOR inhibitor partially decreased the phosphorylated status of P70S6K in both G-415 and TGBC-2TBK tumors. With the WYE-354 regimen, only half of the analyzed tumors, showed a significant but marginally decrease in phosphorylation status of P70S6K, compared to control mice. By contrast, all the tumors from animals treated with rapamycin showed a significant but marginally reduction in phospho-P70S6K. As shown in Supplementary Figure S1 and Supplementary Figure S2 (Figure S1 and Figure S2), an immunohistochemical analysis of phospho-4E-BP1 was performed on the xenograft tumors tissues. We observed that treatments with either mTOR inhibitor partially decreased the phosphorylated status of 4E-BP1 in both G-415 and TGBC-2TBK tumors. Mice treated with rapamycin showed lower expression of phospho-4E-BP1 in both cell lines, G-415 and TGBC-2TKB (Supplementary Figure S1). Mice treated with WYE-354 display regions with lower staining expression and smaller area of phospho-4E-BP1 compared with controls in both cell lines (Supplementary Figure S2). Altogether, the qualitative analysis of the staining of phospho-4E-BP1 showed a decrease in its expression in mice treated with each of the inhibitors in both cell lines. For WYE-354, the effect appears to be more noticeable for the cell line G-415, which is related to the greater cytotoxic effect observed in this cell line compared to TGBC-2TKB (Figure 2. Tumor weight reduction 82.9% vs 45.5%).

We also investigated the Ki67 proliferative index in tumors from animals treated with both mTOR inhibitors at the end of the *in vivo* assays. The expression of Ki67 was determined via standard immunohistochemical techniques on serial paraffin sections. The results showed that the number of Ki67 positive cells was decreased in both G-415 and TGBC-2TKB tumors from mice treated with rapamycin, 58.8% and 28.5%, respectively, (*P* < 0.5; ns) compared with their controls (Figure 3E). The proliferation index in tumors from animals treated with WYE-354 was significantly decreased only in TGBC-2TKB tumors - 23.2%; (*P* < 0.01) - and interestingly showed a higher Ki67 expression in treated G-415 tumors compared to the control, but this difference did not reach significance (Figure 4E).

## DISCUSSION

Deregulation of the mTOR signaling pathway has been associated with the pathogenesis of various human cancers [20, 22, 39–44], and studies have shown its role in angiogenesis, progression, and treatment resistance [45]. In this study, we have evaluated for the first time the therapeutic efficacy of two mTOR inhibitors, rapamycin and WYE-354, in preclinical models of human GBC in NOD-SCID mice.

The best characterized downstream substrates of mTORC1 are the ribosomal protein P70S6K and 4E-BP1. Phosphorylation of 4E-BP1 releases eIF4E, allowing the initiation of translation. By acting on P70S6K, mTOR facilitates ribosome biogenesis and translation elongation [46–51]. As a downstream effector of mTOR pathway, the phosphorylation status of P70S6K commonly is used as a marker of mTOR activity and for the pharmacodynamic monitoring of mTOR inhibition [52, 53]. Previously published data from our group shows that phospho-mTOR and phospho-P70S6K expression are significantly higher in human gallbladder carcinoma than in non-neoplastic tissues and that high expression of phospho-mTOR is associated with poor survival in patients with advanced GBC [33, 34]. Similar to our previous *in vitro* studies with rapamycin in GBC cells [33], WYE-354 inhibited cell viability and decreased cell migration and invasion, accompanied by a markedly decreased phosphorylation status of P70S6K and 4E-BP1 under the treatment conditions assayed. These results indicate that WYE-354 negatively regulated the phosphorylation of effectors downstream from mTOR (mTORC1) and inhibited the mTOR signaling pathway under the study conditions. Although it has been reported that concentrations equal to or greater than 10 nM of WYE-354 have off target-effects on mTOR pathway [38, 54] we did not observe a decrease of *in vitro* cell survival at a dose of 100 nM. Similar observations have been reported for some tumoral cell lines treated with rapamycin. Concentrations of rapamycin required to reduce *in vitro* cell viability in breast cancer cells and malignant glioma cells, were significantly higher than the concentrations needed to suppress levels of phospho-P70S6K and phospho-4E-BP1 [55–58]. However, the efficacy of rapamycin is tumour- and cell-type specific, and the precise mechanism behind its actions is not completely understood [59].

Several studies have demonstrated the therapeutic efficacy of mTOR inhibitors on suppressing tumor growth in a number of solid tumors on human subcutaneous growing xenografts [19, 21, 60] and in many phase I–III clinical studies [16, 17, 32]. However, only a few studies have reported the use of mTOR inhibitors on *in vivo* human GBC. Zong *et al*. [36] used rapamycin to study the effect of mTOR pathway inhibition on migration and invasion of GBC in tumor metastasis mouse models. Weng *et al*. used rapamycin as an immunosuppressant that modified host innate or adaptive cellular immunity, facilitating virus infection for oncolytic virotherapy with myxoma virus, but rapamycin was not used as a treatment itself [61]. Our study demonstrated the high therapeutic efficacy of two mTOR inhibitors on suppressing tumor growth on subcutaneous GBC tumor models. At the regimens and doses studied, both rapamycin and WYE-354, were well tolerated with no significant weight loss or adverse effects, suggesting the toxicity of these inhibitors is low at least under the studied conditions. It is likely that the better therapeutic response observed in mice treated with rapamycin compared to WYE-354-treated mice was strongly related to the treatment regimen. Although both treatments significantly inhibited tumor growth compared to their controls, a prolonged- low dose treatment with rapamycin was clearly more effective at decreasing tumor mass than a short-term treatment with a higher dose of WYE-354. Hu *et al*. [19] observed that the rate of tumor growth in preclinical animal models of head and neck squamous cell carcinoma increased on days without rapamycin treatment. This observation and our results may imply that a continuous a long term treatment may provide better outcomes in inhibiting tumor growth than a short term and cyclical treatment regimen.

It has been widely studied that the positive regulation of AKT by mTORC2 leads to mTOR acting both upstream and downstream from AKT [16, 62]. Unlike WYE-354, rapamycin does not directly inhibit mTORC2, due to the FRB domain on mTORC not being accessible on mTORC2 [63, 64]. However, the remarkable therapeutic response observed in our study with rapamycin treatment may be in part due to the fact that a prolonged rapamycin treatment inhibits mTORC2 in some types of human cancer cells by preventing mTORC2 complex assembly, blocking the phosphorylation of its substrate AKT at Ser473 [51, 60]. The fact that the better anti- tumor effect observed in rapamycin-treated mice was accompanied with a greater decrease in the phosphorylation status of P70S6K suggests a more effective mTOR pathway inhibition [19, 52, 53]. However, both inhibitors only partially inhibited phospho-P70S6K in most tumors treated. This could suggest that the antitumor effect of these drugs is likely to be indirect. Some studies have shown that the effect might be via targeting normal cells [65]. Lkb1+/− mice treated with rapamycin showed a reduction in gastric tumor burden and in the number of polyps per mouse. Yet, the phosphorylation of ribosomal P70S6 kinase in the polyps from the treated mice was maintained. However, a significant reduction in microvessel density was seen in these polyps [59]. Systemic rapamycin treatment of mammary tumors grown in Cav-1 KO mice not only inhibited tumor growth but also stromal content and angiogenesis [66]. Checkley *et al*., reported that rapamycin inhibited skin tumor promotion accompanied with a significantly decrease in the number of infiltrating macrophages, T cells, neutrophils, and mast cells in the dermis [67].

Tumor mass reduction observed in both rapamycin and WYE-354 treated groups was associated with fewer vascularized tumors than in the controls. Several studies have established that the antitumor efficacy of rapamycin and its rapalogs as well as pyrazolopyrimidines such as WYE-354 can be attributed partly to a reduction in HIF1-alpha expression, which is an activator of vascular endothelial growth factor (VEGF) gene transcription [38, 46, 68–71]. In conclusion, our findings provide a proof of concept for the use of mTOR pathway-targeted therapies as potentially good candidates in clinical trials in advanced GBC patients.

## MATERIALS AND METHODS

### Cell culture

The human GBC cells G-415 and TGBC2KB were obtained from Riken BioResource Center (Ibaraki, Japan). G-415 were grown in Roswell Park Memorial Institute (RPMI) 1640 medium and TGBC-2TKB were grown in Dulbecco's Modified Eagle's Medium (high glucose), supplemented with 10% fetal bovine serum, 2 mM glutamine, 100 U/ml penicillin and 100 μg/ml streptomycin and maintained in a 37°C atmosphere containing 5% CO_2_.

### mTOR inhibitors

Rapamycin inhibitor (R-5000) was purchased from LC Laboratories (Woburn, MA, USA) and WYE-354 (A10114) was purchased from Adooq Bioscience LLC (Irvine, CA, USA). For *in vitro* assays the inhibitors were dissolved at 10 mM in dimethylsulfoxide as stock solutions and stored at −20°C. Inhibitors were diluted in culture medium before each *in vitro* experiment and 0.01% dimethylsulfoxide in culture medium was used as a vehicle control. For *in vivo* studies rapamycin was formulated in 4% ethanol, 5.2% Tween 80 and 5.2% PEG 400, while WYE-354 was formulated in 5% ethanol, 5% Tween 80 and 5% PEG 400 and stored at −20°C.

### Antibodies

Rabbit monoclonal antibodies, anti-P70S6K (#2708) and anti-phospho-P70S6K (Thr389, #9234) anti-eIF4E (#2067), anti-phospho-eIF4E (Ser209, #9741), anti-4E-BP1 (#9644), anti-phospho-4E-BP1 (Thr37/46, #2855) and anti-β-actin (#4970) were purchased from Cell Signaling Technology and used for Western blot analysis. The secondary antibody was goat anti-rabbit IgG horseradish peroxidase (Santa Cruz Biotechnology Inc.). Mouse monoclonal antibody anti-Ki67 (Clone MIB1 DAKO, Glostrup, Denmark) was obtained from Dako, Agilent Technologies and rabbit monoclonal antibody anti-phospho-4E-BP1 (Thr37/46, #2855) was purchased from Cell Signaling Technology and used for immunohistochemical staining.

### Western blot analysis

For *in vitro* assay, cells were seeded on 75 cm2 culture plates. At 60–70% confluency the cells were treated with WYE-354 (1 μM), rapamycin (50 nM), and 0.01% dimethylsulfoxide (as control) for 18 hours. Cell lysate was prepared using RIPA buffer (Sigma-Aldrich Co, St Louis, MO, USA). Briefly, cells were washed three times with cold phosphate-buffered saline (PBS) and lysed on ice with RIPA buffer containing a phenylmethylsulfonyl fluoride (PMSF), protease and phosphatase inhibitor cocktail (Sigma-Aldrich Co, St Louis, MO, USA). For *in vivo* experiments, tumor lysates were prepared on ice using cell lysis buffer (1% Triton X-100, 150 mM NaCl, 50 mM Tris-HCl pH 7.4, 2 mM EDTA) containing a protease and phosphatase inhibitor cocktail (Sigma-Aldrich Co, St Louis, MO, USA). Whole lysates were collected after centrifugation at 8,000g for 10 minutes at 4°C. Protein concentrations were determined using a BCA Protein Assay Kit (Pierce, Thermo Fisher Scientific Inc, Rockford, IL, USA) according to the manufacturer's instructions. Equal amounts of total cellular protein (70 μg) were separated by sodium dodecyl sulfate-polyacrylamide gel electrophoresis in 4%–12% NuPAGE^®^ Bis-Tris Precast Gels (Novex, Life Technologies Corporation) and electrotransferred to polyvinylidene difluoride membranes (Immobilon^®^-P membrane Millipore, Bedford, MA, USA). The membranes were blocked with 1 × Tris-buffered saline containing 0.05% Tween (TBST) and 5% fat-free milk for 1 hour at room temperature and incubated overnight at 4°C with primary antibodies. After washing with TBST, the membranes were further incubated with the corresponding horseradish peroxidase-conjugated secondary antibodies for 1 hour at room temperature. Antibody-bound protein bands were detected with enhanced chemiluminescence reagent SuperSignal West Pico Substrate (Pierce). Images were acquired on a Thermo Scientific myECL Imager. β-actin expression was used as a loading control.

### Cell viability assays

G-415 and TGBC-2TKB cell lines were plated onto 96 well plates at a density of 2 × 10^3^ cells per well. After an overnight attachment period cells were treated with WYE-354. The number of viable cells was determined at certain intervals using CellTiter 96 Aqueous One Solution Cell Proliferation assay (Promega Corp., Madison, WI). Briefly, 20 μl CellTiter 96 solution was added to each well and the plates were incubated for 2 hour after which the absorbance of each well was read at a wavelength of 490 nm using a multiwell plate reader (Autobio Labtec Instruments Co, Ltd, Zhengzhou City, People's Republic of China). All assays were performed in quintuplicate, and each assay was repeated three times.

### Transwell cell migration and invasion assays

Migration assays were performed using 24-well Transwell™ plates containing polycarbonate filters with an 8 μm pore size (BD Biosciences, Bedford, MA, USA). Cells were exposed to WYE-354 (1 μM), rapamycin (50 nM) and 0.01% dimethylsulfoxide (as control) for 12 hours at 37°C. Complete medium was placed in the lower chamber to act as a chemoattractant and G-415 or TGBC-2TKB cells were seeded at a density of 2.5 × 10^4^ or 5 × 10^4^ cells, respectively, with serum-free medium into the upper chamber. After 24 hours, the cells were fixed in methanol for 15 minutes and then stained with 0.05% crystal violet in 25% methanol/PBS for 15 minutes. Cells on top of the membrane were removed using a cotton swab, and the filters were washed with PBS. Cells on the underside of the filters were viewed and counted under a microscope in 6 randomly selected fields. Invasion assays were performed as for the migration assays described above, except inserts were pre-coated with Matrigel (Corning^®^ Matrigel^®^ Growth Factor Reduced (GFR) Basement Membrane Matrix, #356230) at concentrations of 20 μg for G-415 and 10 μg for TGBC2-TKB cell lines.

### *In vivo* studies on subcutaneous tumors

8 to 12-week- old NOD-SCID mice (obtained from the animal facility of the Georg Speyer Haus, Frankfurt, Germany) were subcutaneously injected in one flank with either 2 × 10^6^ or 5 × 10^6^ cells of G-415 or TGBC2TKB, respectively, and re-suspended in 200 μl of PBS with 30% of Matrigel (Matrigel™ Basement Membrane Matrix, BD Biosciences). When the average tumor reached 100 mm^3^, mice were randomly separated into four groups and treated with rapamycin or WYE-354 and its respective vehicles. Rapamycin was administered at a daily intraperitoneal (i.p) dose of 10 mg/kg for 5 days per week for 3 weeks, while WYE-354 was administrated at a daily i.p dose of 50 mg/kg for 5 days. Tumor volumes were estimated twice a week from caliper measurements (volume = 0.52 × (width)^2^ × length).

### Ethics statement

Mouse husbandry and animal experiments has been conducted in accordance with the ethical standards and according to the local animal protection law.

### Immunohistochemical staining

Xenograft tumors were freshly removed, fixed in 10% buffer formalin and subsequently embedded in paraffin. The sections were manually stained with hematoxylin and eosin (HE) for normal histological evaluation. 3 μm-thick sections were deparaffinized with Histo-Clear (National Diagnostics; Atlanta, GA, USA) and rehydrated through alcohol gradient. Heat-induced antigen retrieval was performed by immersing slides in Tris/EDTA pH 9.0 buffer (10 mMTris, 1 mM EDTA) for 20 min at 99°C in a thermo-regulated bath.

Automated staining was performed using an antibody against Ki67 antigen (Clone MIB1 DAKO, Glostrup, Denmark) at a dilution of 1:100. Ki67 index was measured using the open access web application ImmunoRatio for automated image analysis [40] using 3–10 images per sample, depending on tumor size and excluding necrotic areas. Statistical analyses were performed using an unpaired Mann Whitney test using GraphPad Prism v. 5 (GraphPad Software, Inc. San Diego CA).

An immunohistochemical analysis of phospho-4EBP1 was performed on the xenograft tumors tissues using the antibody Phospho-4E-BP1 (Thr37/46) (236B4) Rabbit mAb #2855 (Cell Signaling Technology) at a dilution of 1:400. All samples were processed simultaneously under the same conditions using an autostainer.

### Statistical analysis

Statistical analyses of *in vitro* and *in vivo* experiments were performed by analysis of variance (one way ANOVA) followed by Tukey's multiple comparisons test. *P* values less than 0.05 were considered significant. Data analysis was performed with the GraphPad Prism 5 (GraphPad Software, Inc. San Diego CA).

## SUPPLEMENTARY MATERIALS FIGURES


