# Supplementary material for: Rapamycin and WYE-354 suppress human gallbladder cancer xenografts in mice
Source: Oncotarget. 2015 Sep 11;6(31):31877–88. doi: 10.18632/oncotarget.5047 (PMC4741647; doi:10.18632/oncotarget.5047)
Supplement: Supplementary file 1 [file oncotarget-06-31877-s001.pdf]

SUPPLEMENTARY FIGURES

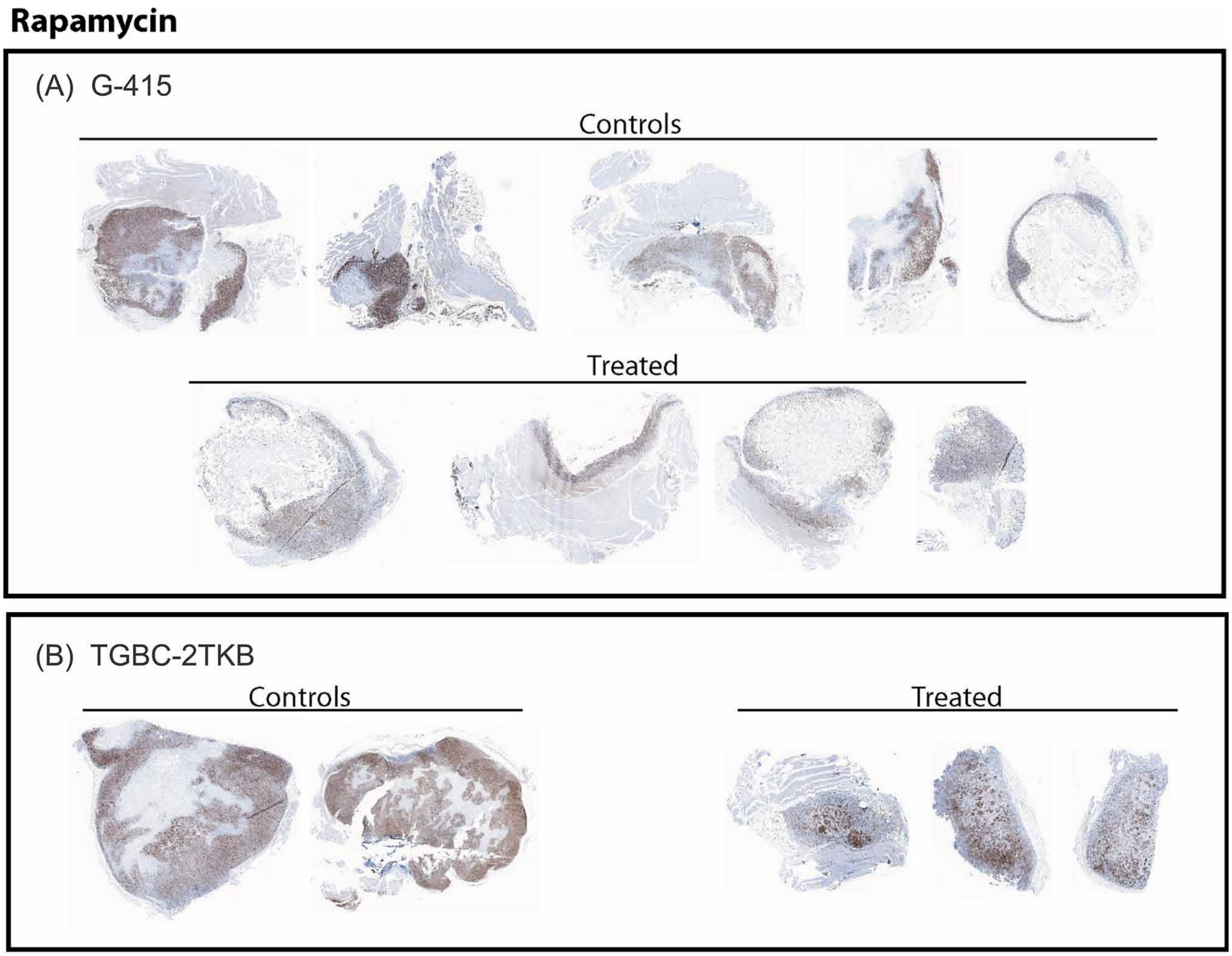

Supplementary Figure S1: Immunohistochemical staining of phospho-4E-BP1 in GBC xenografts treated with rapamycin. A. Staining of phospho-4E-BP1 in G-415 tumors. **B.** Staining of phospho-4E-BP1 in TGBC-2TKB tumors. Images represent original magnification 20×.

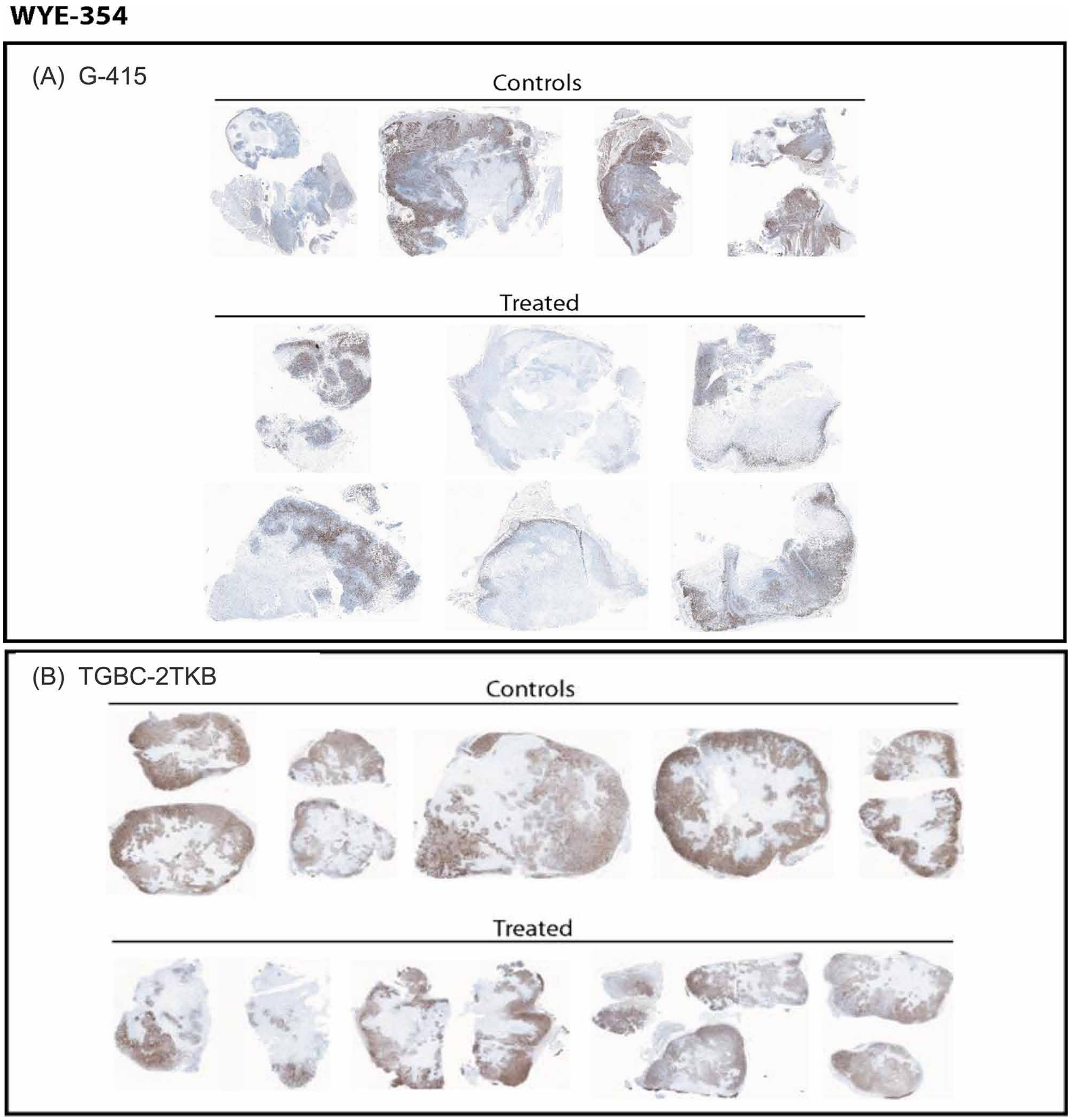

Supplementary Figure S2: Immunohistochemical staining of phospho-4E-BP1 in GBC xenografts treated with WYE-354. A. Staining of phospho-4E-BP1 in G-415 tumors. **B.** Staining of phospho-4E-BP1 in TGBC-2TKB tumors. Images represent original magnification 20×.
